# Quantification of silver nanoparticle toxicity to algae in soil via photosynthetic and flow-cytometric analyses

**DOI:** 10.1038/s41598-017-18680-5

**Published:** 2018-01-10

**Authors:** Sun-Hwa Nam, Jin Il Kwak, Youn-Joo An

**Affiliations:** Department of Environmental Health Science, Konkuk University, 120 Neungdong-ro, Gwangjin-gu, Seoul, 05029 Korea

## Abstract

Soil algae, which have received attention for their use in a novel bioassay to evaluate soil toxicity, expand the range of terrestrial test species. However, there is no information regarding the toxicity of nanomaterials to soil algae. Thus, we evaluated the effects of silver nanoparticles (0–50 mg AgNPs/kg dry weight soil) on the soil alga *Chlamydomonas reinhardtii* after six days, and assessed changes in biomass, photosynthetic activity, cellular morphology, membrane permeability, esterase activity, and oxidative stress. The parameters measured were markedly affected by AgNP-induced stress at 50 mg AgNPs/kg dry weight soil, where soil algal biomass, three measures of photosynthetic activity (area, reaction center per absorption flux, and reaction center per trapped energy flux), and esterase activity decreased. AgNPs also induced increases in both cell size and membrane permeability at 50 mg AgNPs/kg dry weight soil. In addition to the increase in cell size observed via microscopy, a mucilaginous sheath formed as a protective barrier against AgNPs. Thus, the toxicity of AgNPs can be effectively quantified based on the physiological, biochemical, and morphological responses of soil algae, where quantifying the level of toxicity of AgNPs to soil algae could prove to be a useful method in terrestrial ecotoxicology.

## Introduction

Silver nanoparticles (AgNPs) have been used in a wide variety of consumer products (e.g., anti-microbial coatings, textiles, paints, cosmetics, cleaning agents, plastics, medtech, and consumer electronics)^1–3^. Their production has been estimated as 2.8–20 tons/year in the USA, 5.5 tons/year in Europe, and 55 tons/year worldwide^3,4^. Owing to the continuous production and use of AgNPs, their release into the natural environment has increased, where the concentration of AgNPs in soil has been estimated to be 0.02–0.1 μg/kg^2^, 0.007–0.03 μg/kg in the USA, and 0.02–0.06 μg/kg in Europe^5^. Thus, AgNPs accumulated in soil may present a risk to living terrestrial organisms as a result of the ecotoxicity of discrete AgNPs, agglomerated or aggregated AgNPs, transformed AgNPs to silver sulfide (Ag_2_S), or Ag ions released from the AgNPs^3,6,7^. Despite the obvious interest and importance of assessing the toxicity of AgNPs in soil, relatively few studies have provided data regarding their toxicity in actual terrestrial soil systems. Previous investigations of AgNP toxicity have included observations of the inhibition of growth inhibition in plants^8,9^; effects on the feeding, survival, or the inhibition of reproduction in arthropods^10,11^; the inhibition of reproduction and significant changes in gene expression in oligochaete earthworms^12^; and the inhibition of the activities of exoenzymes^13^. Soil algae, which are major primary producers as well as a source of food^14^, were utilized in a new bioassay^15–17^ intended to expand the variety of test species used to assess soil toxicity. Although the toxicity of AgNPs has been evaluated using freshwater and marine water algae^18–21^, to the best of our knowledge, there are no studies that focus on the effects of nanomaterials on soil algae in soil media. Studies on marine and freshwater algae have included observations of inhibited viability and superoxide production in the marine raphidophyte *Chattonella marina* ^18^, the inhibition of growth in the freshwater chlorophyte *Pithophora oedogonia* and *Chara vulgaris* ^19^, the inhibition of the antioxidant potential in the freshwater chlorophyte *Acutodesmus dimorphus* ^20^, and the inhibition of photosynthesis in the freshwater chlorophyte *Chlamydomonas reinhardtii* ^21^. Several parameters have been measured in algal bioassays to assess the effects of potential toxins, including growth inhibition (cell counts, cell volume, fluorescence, optical density, and dry weight), photosynthesis, biomass composition (e.g., lipids, carbohydrates, proteins, and nucleic acids), cellular enzyme activity, cell viability, and cell membrane integrity^22^. Thus, while it is possible to assess the potential toxicity of various substances in algae using liquid media, the potentially toxic effects of nanoparticles on soil algae in soil media have not yet been explored.

Here, we provide a quantitative assessment of the effects of AgNPs on the biomass, photosynthetic activity, cellular morphology, membrane permeability, esterase activity, and oxidative stress of a representative soil alga, *Chlamydomonas reinhardtii* ^23^, in soil media. This approach could provide both a powerful means of evaluating the potential toxicity of nanomaterials based on the response of soil algae, and a quantitative indication of the toxicity of AgNPs in soil.

## Methods

### Test species and pre-culture

*Chlamydomonas reinhardtii* was acquired from the University of Texas, Austin, USA. Algal cells were incubated in tris-acetate-phosphate medium, using 250 mL borosilicate glass flasks with air-permeable stoppers, and sub-cultured at 24 ± 2 °C, with shaking at 100 r/min and a 16:8 h (light:dark)-photoperiod, provided by cool-white fluorescent lamps (~3720 lx).

### Reagents

Powdered AgNPs (<100 nm particle size; 0.2% polyvinylpyrrolidone as a dispersant) were purchased from Sigma-Aldrich (Saint Louis, MO, USA). A field emission transmission electron microscope (FE-TEM; TECNAI G^2^ F30 ST, 300 kV; FEI Co., Hillsboro, OR, USA) was used to observe the morphology of AgNPs. A surface area analyzer (Microtrac, Montgomeryville, PA, USA) was used to observe the surface area of some AgNPs. Field emission scanning electron microscopy (FE-SEM; SUPRA 55VP; Carl Zeiss, Jena, Germany) with an energy-dispersive X-ray spectroscopy detector (EDX; XFlash detector 5010; Bruker, USA) was used to observe the distribution and level of AgNPs in 50 mg AgNPs/kg (dry weight) soil. To compare the effects of bulk and ionic Ag with those of AgNPs, bulk Ag powder (2–3.5 μm particle size; 99.9% trace metals basis) and silver nitrate powder (AgNO_3_; 99%) were purchased from Sigma-Aldrich. AgNO_3_ was dissolved in autoclaved deionized water to prepare 1,000 mg Ag/L as a stock solution, and serial dilutions of ionic Ag were prepared with autoclaved deionized water for treatments.

### Test soil and spiking procedure of AgNPs, bulk Ag, and ionic Ag

A natural and commercially available LUFA 2.2 soil (LUFA-Speyer, Sp 2121, Speyer, Germany) was used as the test soil. The physicochemical properties of LUFA 2.2 soil are presented in Table S1. Powdered AgNPs were spiked in autoclaved (15 min, 121 °C) LUFA soil at the nominal concentration of 50 mg AgNPs/kg dry weight, and thoroughly mixed on a roller (40 r/min) for 24 h. Then, 50 mg AgNPs/kg (dry weight) was diluted in untreated soil, to provide the concentrations of 10, 20, 30, and 40 mg AgNPs/kg (dry weight). To compare bulk and ionic Ag with AgNPs, bulk Ag-treated soil was prepared by the same process as aforementioned for the preparation of AgNPs. To measure the dissolution of AgNPs in soil after six days, deionized water was added to the maximum concentration of test soil (2:1 ratio of water:soil) and thoroughly mixed by vigorous and repeated pipetting. The slurry was filtered through mixed-cellulose-ester syringe filters (0.45 μm porosity and 25 mm diameter; Advantec) and an ultrafiltration membrane (nominal molecular mass limit 30 kDa, Model 8050; Millipore, USA). The filtrates were measured by inductively coupled plasma-mass spectrometer (ICP-MS; Elan DRC II, Perkin Elmer, Norwalk, CT, USA; 0.01 μg/L of detection limit). Although no Ag ions were detected to have been released from AgNP-treated soil, algal toxicity for ionic Ag was performed at 5, 10, 20, 30, 40, and 50 mg Ag/kg (dry weight), as the dissolution of AgNPs was conducted in diluted soil extracts after the addition of deionized water.

### Exposure of soil algae to soils with AgNPs, bulk Ag, or ionic Ag

The potential toxicity of AgNPs to the soil alga *C*. *reinhardtii* was performed according to the modified protocol of Nam and An^17^. Samples (1 g) of soil were transferred to a flat-bottom 6-well microplate (diameter 35 mm, height 22.5 mm for each well). Deionized water was added to adjust the moisture content of the test soil to 90% of its water holding capacity. Once the test soil was saturated with deionized water, 0.13 mL of *C*. *reinhardtii* during exponential growth (i.e., initial density of 6.5 × 10^5^ cells/g in the test soil), was inoculated onto the soil surface of triplicate samples. Simultaneously, 0.13 mL of deionized water (instead of algal suspension) was added to the wells in the same manner to correct for background fluorescence in the biomass analysis or to exclude the effects of solid fine soil particles in the flow cytometry. Microplates were incubated for six days under the same pre-culture conditions under which *C*. *reinhardtii* were reared, but without shaking. In addition, to evaluate the toxicity of ionic Ag, untreated soil and serial solutions of ionic Ag were added to the wells in the same manner as treatments for AgNPs or bulk Ag-treated soil and AgNPs. Likewise, deionized water was used in place of ionic Ag in control samples.

### Extraction of soil algae from AgNPs, bulk Ag, or ionic Ag-soils

To extract intact *C*. *reinhardtii* grown in each soil treatment, 5 mL of Bold’s Basal Medium (BBM) was added to each soil sample, and the microplate was then shaken for 24 h under pre-culture conditions. The resulting supernatant was used to analyze biomass, photosynthesis, and flow cytometry after a 5-min settling period.

### Biomass analyses

To extract chlorophyll *a* from *C*. *reinhardtii*, an algal suspension was mixed with ethanol (1:4 ratio of algae:ethanol) and shaken in the dark for 3 h under pre-culture conditions. Chlorophyll *a* fluorescence was measured to quantify algal biomass using a fluorescence microplate reader (Gemini; Molecular Devices, Sunnyvale, CA, USA) at an excitation wavelength of 420 nm and an emission wavelength of 671 nm^24^.

### Photosynthetic analyses

To analyze the photosynthetic capacity of *C*. *reinhardtii*, an algal suspension was allowed to adapt to the dark for 15 min. The Handy Plant Efficiency Analyzer (PEA; Hansatech Instruments Ltd., Kings Lynn, Norfolk, UK) was then used to analyze Photosystem II parameters: total complementary area (area), maximum quantum yield of primary photochemistry (at t = 0) (Fv/Fm), reaction center per absorption flux (RC/ABS), reaction center per trapped energy flux (at t = 0) (RC/TRo), electron transport flux per reaction center (at t = 0) (ETo/RC), and reaction center per dissipated energy flux (at t = 0) (RC/Dio)^25,26^.

### Flow cytometry

To analyze cell morphology, membrane permeability, esterase activity, and oxidative stress in *C*. *reinhardtii*, an algal suspension was subjected to flow cytometry (FACScalibur; BD Biosciences, NJ, USA) and data were analyzed using Flowjo V10 software (FlowJo LLC., Ashland, OR, USA). Before analyzing the flow cytometric endpoints, a gating region was determined (as shown in Fig. S1) to separate pure *C*. *reinhardtii* populations from unspecific debris in soil extracts. Data were collected from 10,000 events in the gated *C*. *reinhardtii* population. Cell size and cell granularity were acquired from FSC (forward scattered light) and SSC (side scattered light) signals, respectively. Membrane permeability, esterase activity, and oxidative stress were determined by FL1 (500–560 nm band pass filter, excitation at 488 nm blue laser, 15 mW, argon ion laser) after centrifugation (3000 r/min, 10 min), re-suspension (3 mL of BBM), staining with fluorescein diacetate (FDA), calcein-acetoxymethyl ester (calcein-AM), or 2′,7′-dichlorofluorescin diacetate (DCFH-DA), respectively, as follows. Non-fluorescent FDA, calcein-AM, and DCFH-DA were obtained from Sigma-Aldrich. Each stock solution of dye was prepared as follows: FDA powders were dissolved in acetone (DUKSAN, Seoul, South Korea) to obtain 11 mM solutions, calcein-AM and DCFH-DA powders were dissolved in dimethyl sulfoxide (DUKSAN) to obtain 500 µM solutions, and each stock solution was stored at −50 °C until use. Subsequently, *C*. *reinhardtii* cells were stained with FDA at a final concentration of 0.11 mM for 20 minutes (dark, 20 °C)^27^, stained with calcein-AM at final concentration of 10 µM for 30 min (dark, 37 °C)^28^, or stained with DCFH-DA at final concentration of 10 µM for 30 minutes (dark, 20 °C)^29^. In parallel, unstained groups (autofluorescence) were incubated under the same conditions, as a previously published study^30^ found that FDA and calcein-AM were not applicable to algae owing to the nature of their cell walls. After incubation, the intensities of cellular fluorescein that penetrated the cell membrane^31^, calcein (green fluorescence) hydrolyzed by esterase^28,32^, and dichlorofluorescin (green fluorescence) oxidized by hydrogen peroxide^33^ were evaluated. For quantitative analysis, the geometric means of the FSC, SSC, fluorescein, calcein, dichlorofluorescin, and autofluorescence intensities were normalized and averaged across three to six replicates.

### Microscopic analyses

All samples were prepared in the same manner as the exposure of soil algae to AgNP, bulk Ag, or ionic Ag-soils. After the end of the experiment, the shape of algal cells was analyzed using light fluorescence microscopy (BX-51; Olympus, Tokyo, Japan). To obtain clear images of the algal mucilaginous sheath, samples were negatively stained with nigrosin, based on a method modified from Guedes *et al*.^34^. Nigrosin solution (100 g/L, formalin 5 mL/L in water) was purchased from Sigma-Aldrich. A drop of algal suspension was placed near the edge of a glass slide and mixed gently with a drop of nigrosin. A smear was drawn and dried for 1 sec. Using this method, the algal mucilaginous sheath is unstained (white) while the background becomes black when using a light microscope. For the FE-SEM analysis of algal cells adsorbed to soils or suspended in soil extracts, algal cells exposed to untreated soil and NP-treated soils were centrifuged, and an algal pellet was fixed with 4% glutaraldehyde, and subsequently washed with phosphate buffered saline. The dehydration of the algal pellet was conducted with 50, 70, 80, 90, or 100% ethanol and isoamyl acetate. After gold coating the algal pellet, FE-SEM was used to observe the morphology of algal cells adsorbed to soils or suspended in soil extracts. The analysis of algal cells suspended in soil extracts was performed using a field emission transmission electron microscope (FE-TEM). For this analysis, algal cells that had been exposed to untreated and NP-treated soils were centrifuged, and the resulting algal pellet was processed via fixation, dehydration, embedding, and scission as follows. Fixation was performed using 2.5% glutaraldehyde, 1% osmium tetroxide, and phosphate-buffered saline. Dehydration was performed using 50, 70, 80, 90, and 100% ethanol and 100% propylene oxide. Embedding was performed using a series of a mixture of propylene oxide and epon (2:1, 1:1, 1:2, and 0:1), and polymerization was performed for 72 h. Subsequently, the embedded algal samples were cut into ultra-thin sections (100 nm thickness). The resulting sections of algal cells were analyzed by FE-TEM (JEM-2100F, JOEL Ltd., Japan), with energy dispersive x-ray analysis (EDX) (X-max; Oxford instruments, UK) to observe the distribution and level of AgNPs. Here, algal cells adsorbed to soils were not analyzed using FE-TEM owing to potential damage to the device as a result of soil particles.

### Data analyses

The percent biomass, photosynthesis, cell size, cell granularity, esterase activity, oxidative stress, and membrane permeability for each concentration of AgNPs, bulk Ag, and ionic Ag were normalized to the control group. Data were analyzed using Dunnett’s test and differences at *p* < 0.05 were considered statistically significant. Effective concentrations at 10% (EC_10_) and 50% (EC_50_) were calculated using Probit (Probit Software LTD, USA).

## Results and Discussion

### Characterization of AgNPs

According to the FE-TEM image in Figure S2, AgNPs exhibited an irregular morphology, and an average surface area of 1.8498 ± 0.0191 m^2^/g. According to the SEM image and EDX spectrum displayed in Figure S3, soil surfaces in 50 mg AgNPs/kg (dry weight) soil displayed electron-dense spots corresponding to Ag in comparison to the normal soil matrix.

### Effects of AgNP-soils on soil algal biomass

**Figure 1:**
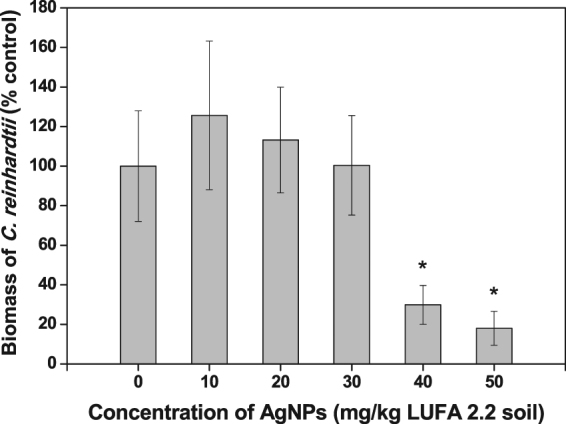
Biomass of *Chlamydomonas reinhardtii* after exposure to silver nanoparticles (AgNPs) for six days. Bars represent the standard deviation of the mean of six replicates. Asterisks (*) indicate significantly different values in relation to those obtained in the control (*p* < 0.05).

Figure 1 illustrates the biomass of *C*. *reinhardtii* in AgNP-treated soils, showing that AgNPs inhibited the growth of *C*. *reinhardtii* at soil concentrations of 40 and 50 mg AgNPs/kg soil in comparison to the control. To date, there have been no studies that have analyzed chlorophyll *a* levels to quantify algal biomass in algae exposed to AgNP-treated soil. As for previous studies of AgNP toxicity using AgNP-treated liquid media, relatively few studies reported that growth in *C*. *reinhardtii* was inhibited in response to citrate-coated AgNPs^35^ and polymer-coated AgNPs^36^.

### Effects of AgNP-soils on soil algal photosynthesis

**Figure 2:**
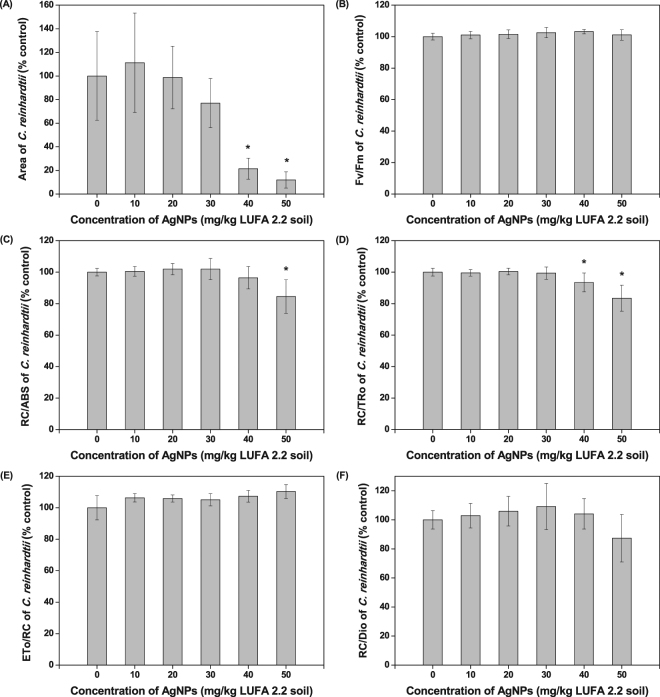
Photosynthetic activity of *Chlamydomonas reinhardtii* after exposure to silver nanoparticles (AgNPs) for six days. Bars represent the standard deviation of the mean of six replicates. (**A**) Total complementary area (Area), (**B**) Maximum quantum yield of primary photochemistry (at t = 0) (Fv/Fm), (**C**) Reaction center per absorption flux (RC/ABS), (**D**) Reaction center per trapped energy flux (at t = 0) (RC/TRo), (**E**) Electron transport flux per reaction center (at t = 0) (ETo/RC), and (**F**) Reaction center per dissipated energy flux (at t = 0) (RC/Dio). Asterisks (*) indicate results that are significantly different from those of the control (*p* < 0.05).

Figure 2A–F show the photosynthetic activity of *C*. *reinhardtii* in AgNP-treated soils. The AgNPs decreased the area, RC/ABS at 50 mg AgNPs/kg treated soil, and RC/TRo of *C*. *reinhardtii* at 40 and 50 mg AgNPs/kg treated soil in comparison to the control (*p* < 0.05), thereby reducing the transfer of electrons from the reaction center to the quinone pool^37^, the inactivation of the reaction center^38,39^, and contributing to a greater reduction in Q_A_ to Q_Ā_ in Photosystem II^39^. The area was the most sensitive endpoint among the photosynthetic endpoints (Table 1). While algal photosynthesis had not been assessed for AgNP-treated soils in previous studies, some studies reported the photosynthetic inhibition of *C*. *reinhardtii* in response to carbonated-coated AgNPs^1^, nine types of coated AgNPs^21^, and other AgNPs^40,41^ in AgNP-treated liquid media.

**Table 1:** Evaluation endpoints of the soil alga *Chlamydomonas reinhardtii* exposed to silver nanoparticles (AgNPs) for six days.

Endpoints^a^		*C*. *reinhardtii*		
		Mean EC_10_ (Min–Max)	Mean EC_50_ (Min–Max)	NOEC^b^
Biomass		32 (5–37)	40 (28–53)	30
Photosynthetic activity	Area	26 (24–27)	35 (34–37)	30
	Fv/Fm	>50	>50	>50
	RC/ABS	46 (43–51)	>50	40
	RC/TRo	44 (40–48)	>50	30
	ETo/RC	>50	>50	>50
	RC/Dio	>50	>50	>50
Cell size		47 (37–81)	>50	30
Cell granularity		>50	>50	>50
Membrane permeability		11 (3–17)	24 (15–32)	20
Esterase activity		Not calculated due to inconsistent staining		
Oxidative stress		Not calculated due to inconsistent staining		

^a^Unit: mg/kg of dry weight soil.

^b^Differences at *p* < *0*.*05* for Dunnett’s test.

### Effects of AgNP-soils on cell morphology

**Figure 3:**
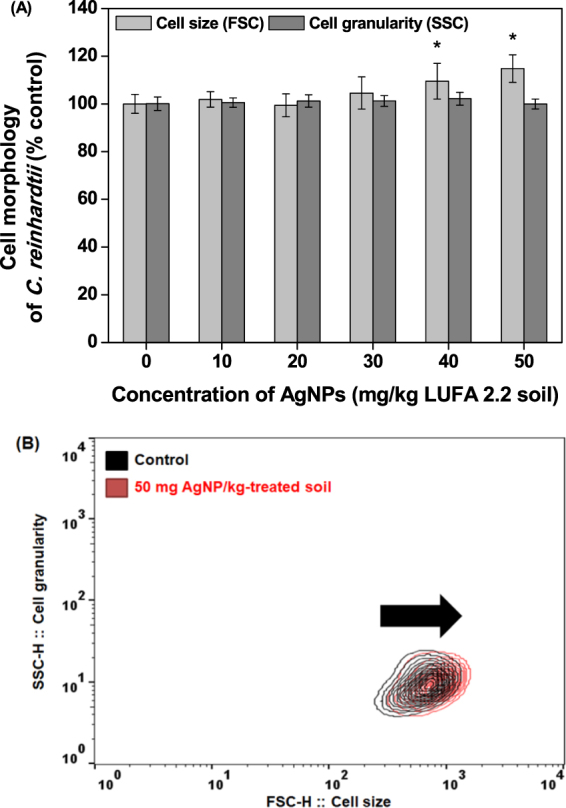
Cell morphology (size and granularity) of *Chlamydomonas reinhardtii* after exposure to silver nanoparticles (AgNPs) for six days. Bars represent the standard deviation of the mean (n = 6–12). (**A**) Flow cytometry histogram and (**B**) Flow cytometry contour plot. Asterisks (*) indicate results that are significantly different from those of the control (*p* < 0.05).

**Figure 4:**
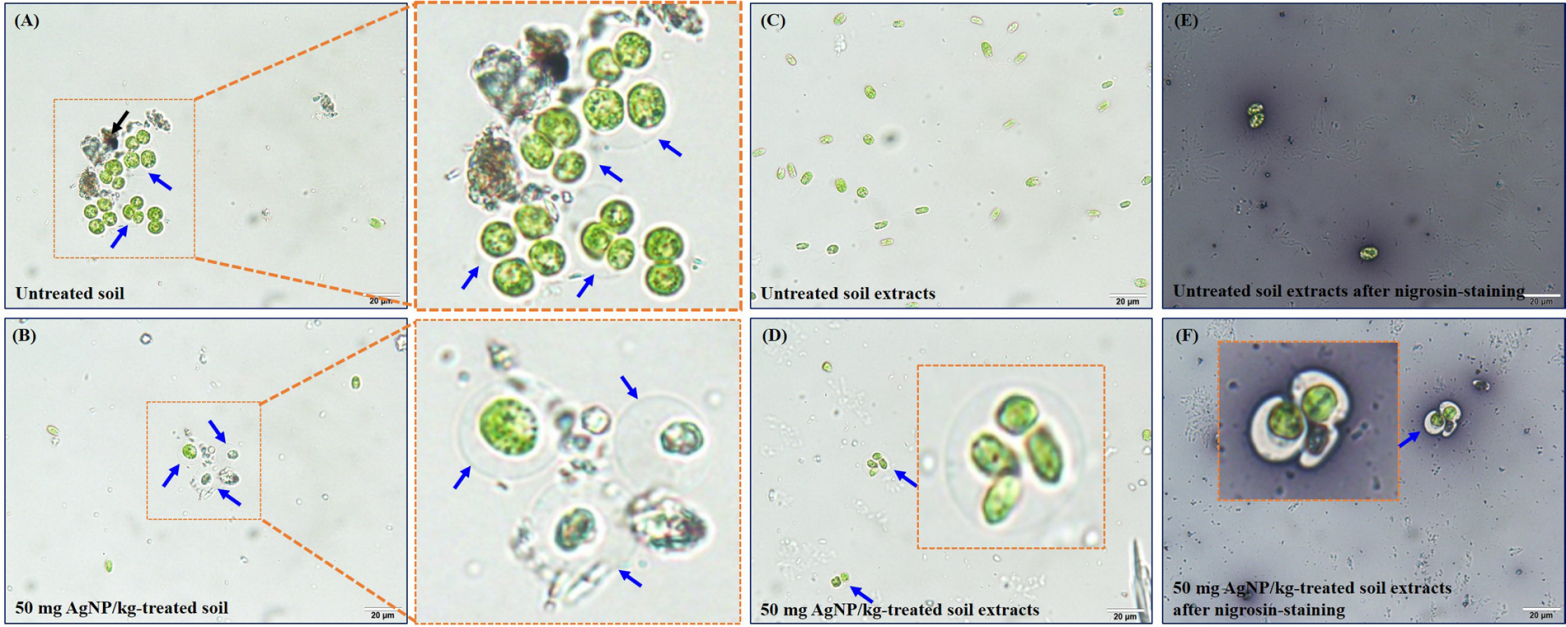
Light micrographs of *Chlamydomonas reinhardtii* after exposure to silver nanoparticles (AgNPs) for six days. (**A**) *C*. *reinhardtii* adsorbed to the untreated soil, (**B**) *C*. *reinhardtii* adsorbed to the 50 mg AgNP/kg-treated soil, (**C**) *C*. *reinhardtii* suspended in the untreated soil extracts, (**D**) *C*. *reinhardtii* suspended in the 50 mg AgNP/kg-treated soil extracts, (**E**) *C*. *reinhardtii* suspended in the untreated soil extracts after nigrosin-staining, and (**F**) *C*. *reinhardtii* suspended in the 50 mg AgNP/kg-treated soil extracts after nigrosin-staining. Black arrows indicate soil particles. Blue arrows indicate mucilaginous sheaths.

**Figure 5:**
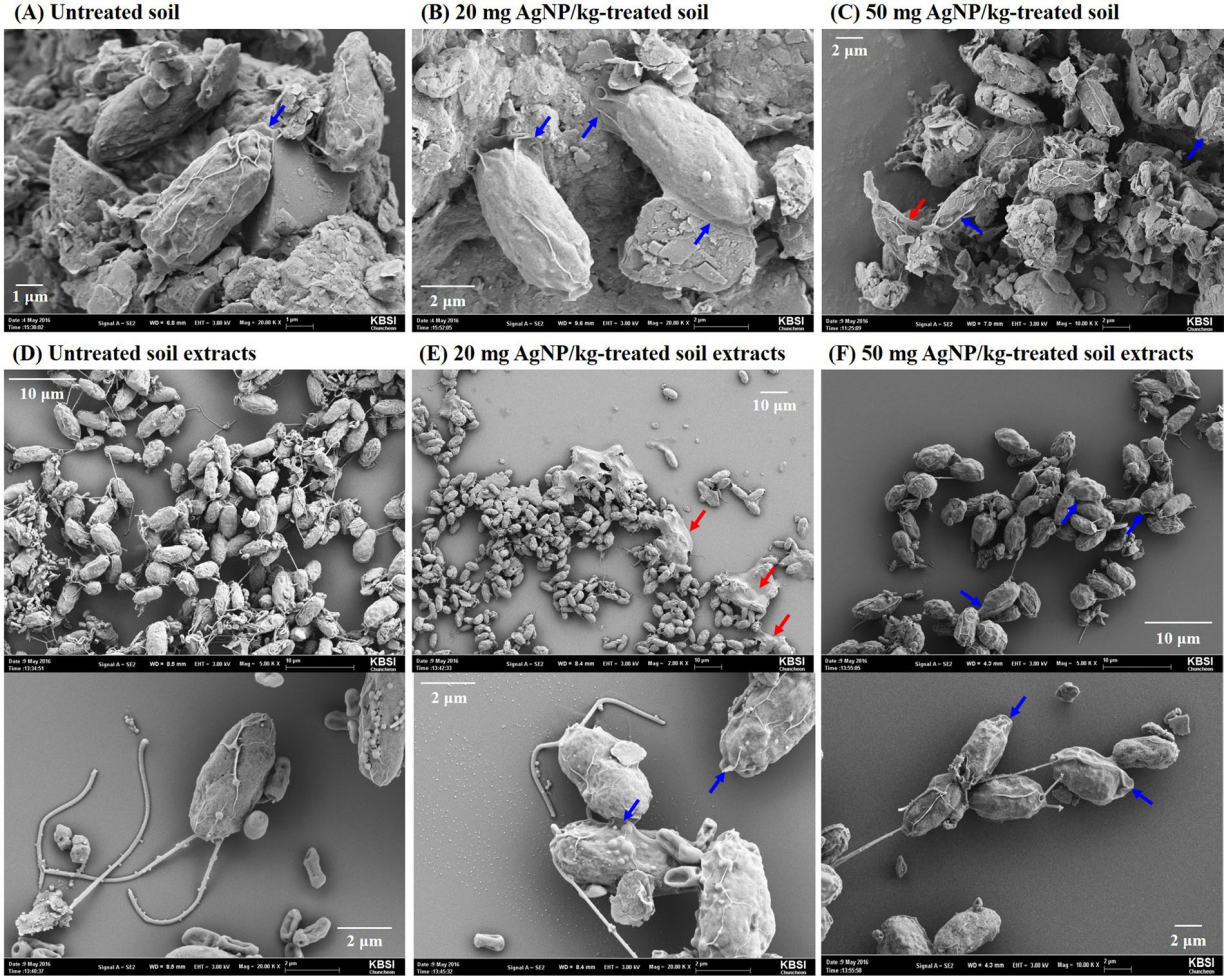
Field emission scanning electron microscopic images of *Chlamydomonas reinhardtii* after exposure to silver nanoparticles (AgNPs) for six days. (**A**) *C*. *reinhardtii* adsorbed to the untreated soil, (**B**) *C*. *reinhardtii* adsorbed to the 20 mg AgNP/kg-treated soil, (**C**) *C*. *reinhardtii* adsorbed to the 50 mg AgNP/kg-treated soil, (**D**) *C*. *reinhardtii* suspended in the untreated soil extracts, (**E**) *C*. *reinhardtii* suspended in the 20 mg AgNP/kg-treated soil extracts, and (**F**) *C*. *reinhardtii* suspended in the 50 mg AgNP/kg-treated soil extracts. Blue arrows indicate mucilaginous sheaths produced from the algal cell wall and red arrows indicate fragments of mucilaginous sheath.

**Figure 6:**
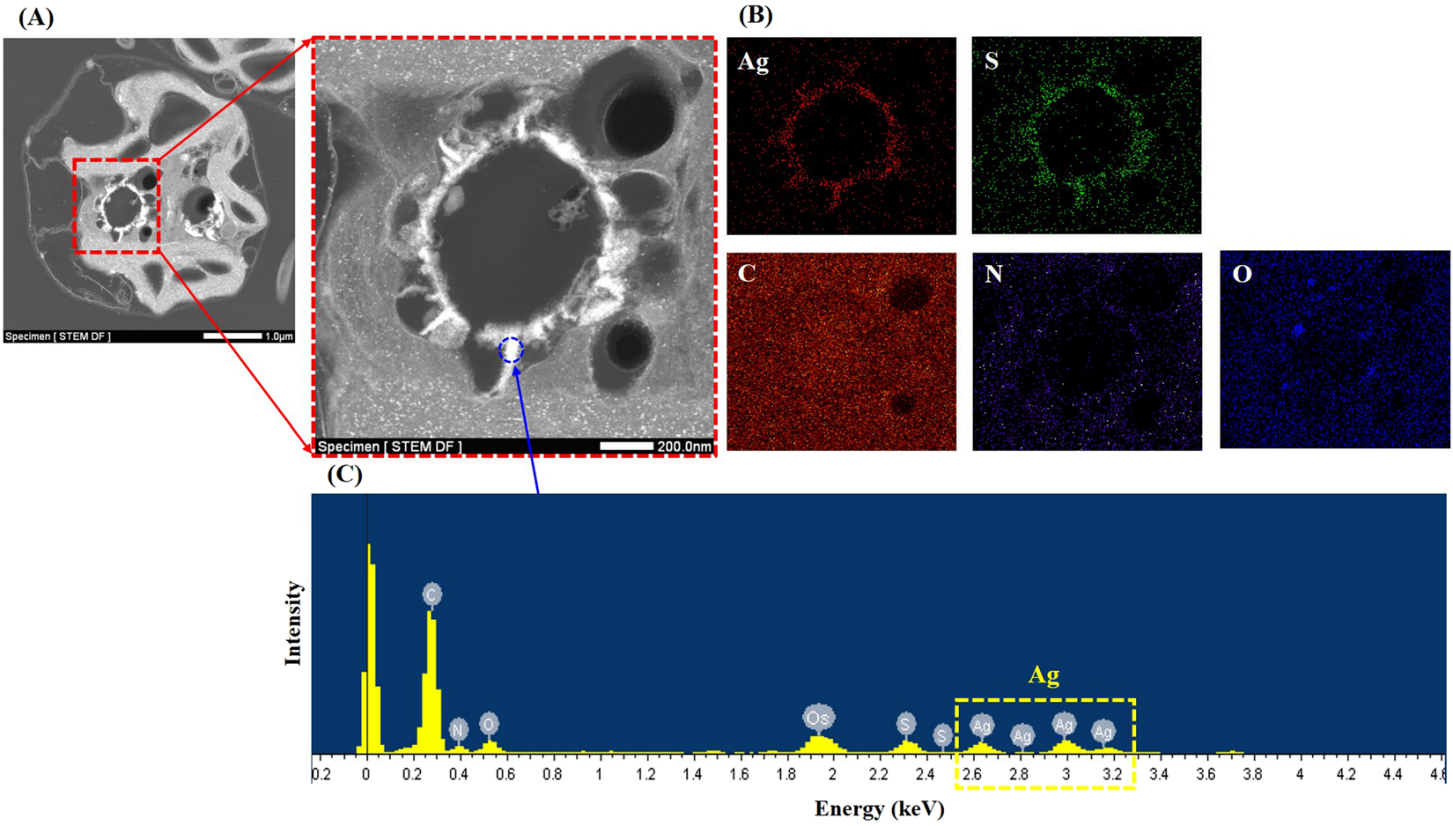
Field emission transmission electron micrographs (FE-TEM) and energy-dispersive X-ray spectroscopy detector (EDX) images of *Chlamydomonas reinhardtii* in 50 mg AgNP/kg-treated soil. (**A**) FE-TEM. (**B**) EDX images show the elemental distribution of Ag, S, C, N, and O in the red dotted square outlining the expanded FE-TEM micrograph from panel A. (**C**) EDX spectrum shows the elemental analysis (C, N, O, S, and Ag, as indicated by labeled blue circles above the yellow peaks). The yellow dotted rectangle indicates the presence of Ag in *C*. *reinhardtii*.

Figure 3 shows the cellular morphology of *C*. *reinhardtii* in response to AgNP-treated soils. The FSC and SSC signals provide cellular morphological parameters that indicate cell size and granularity, respectively. The SSC signal did not change, even at the maximum concentration (50 mg AgNPs/kg), while the FSC signal increased when algae were exposed to ≥ 40 mg AgNP/kg treated soil, indicating an increase in cell size induced by AgNPs. This phenomenon may result from the formation of a mucilaginous sheath around the algal cells, most likely as protective barriers against AgNPs (Fig. 4). In the control group, *C*. *reinhardtii* embedded within a mucilaginous sheath were adsorbed to the soils as non-motile aplanospores and aplanosporangia. In the experimental group, the mucilaginous sheath of 50 mg AgNP/kg-treated *C*. *reinhardtii* cells became thicker than in control cells, while color weakening (green to black) and necrosis were observed in some *C*. *reinhardtii* samples treated with 50 mg AgNPs/kg. Under unfavorable conditions, algal cells excrete exopolysaccharides into the surrounding soil as protection against harmful substances (e.g., metals) and unfavorable conditions (e.g., desiccation), or to provide nutrients and moisture^42,43^, where cells are embedded in a mucilaginous sheath formed by the gelatinization of the parent cell wall (known as the palmelloid stage). In this stage, the mucilaginous sheath plays an important role as a protective barrier against desiccation in sub-terrestrial habitats^44^, predators^45^, chemicals^46–49^, and nutrient deficiency^50^. The palmelloid stage of *C*. *reinhardtii* has been reported as a stress-inducible response, based on exposure to various organic acids^46^, phosphate-limitation^50^, rotifer predators^45^, paraquat^47^, and sodium chloride^48,49^. In the present study, based on the observation that algal cells in the AgNP-treated soils exhibited thicker mucilaginous sheaths than those in untreated soils, the mucilaginous sheath could be induced in untreated and AgNPs-treated damp soils as a result of desiccation, while the changes in the thickness of the mucilaginous sheath observed at high concentrations of AgNPs could be an AgNP-inducible response. In addition, the mucilaginous sheath of *C*. *reinhardtii* cells suspended in untreated soil extracts disappeared, and motile zoospores and zoosporangia with flagella appeared (Fig. 4C). This could have been induced by the natural migration of non-motile cells from soil into the BBM, and the extinction of the mucilaginous sheath as a protective barrier, where the natural transformation of non-motile cells to motile cells could be induced under favorable conditions (namely, the 1 d-extraction with liquid media in this study). In contrast, the mucilaginous sheaths of *C*. *reinhardtii* suspended in the 50 mg AgNP/kg-treated soil extracts were partially maintained after the 1 d-extraction with BBM, as described in Fig. 4D. While Fig. 4E,F show sufficient contrast to clearly define the mucilaginous sheath of *C*. *reinhardtii* using the nigrosin-staining method, it was not possible to confirm the presence of a mucilaginous sheath in the untreated soil extracts (Fig. 4E). However, a clearly visible mucilaginous sheath was observed in the 50 mg AgNP/kg-treated soil extracts (Fig. 4F). This mucilaginous sheath could be maintained as a protective barrier in the face of high concentrations of AgNPs, although they were in favorable liquid media. Figure 5A–C show the morphology of *C*. *reinhardtii* adsorbed to untreated and to 20 and 50 mg AgNP/kg-treated soils using HR-SEM, while Figure 5D–F show the external morphology of *C*. *reinhardtii* suspended in untreated and in 20 and 50 mg AgNP/kg-treated soil extracts using HR-SEM. As described in Fig. 4, the mucilaginous sheath produced from the algal cell wall was observed in HR-SEM images, except in untreated soil extracts, where fragments of mucilaginous sheaths were observed. Algal flagella appeared in all soil extracts (Fig. 5), and some fine soil particles were adsorbed to the flagella. Figures S4A and 6 show the internal morphology of *C*. *reinhardtii* suspended in untreated and 50 mg AgNP/kg-treated soil extracts using FE-TEM. We verified the distribution of Ag in algal cells suspended in 50 mg AgNP/kg-treated soil extracts using EDX (Fig. 6B), where Ag was detected in the cytoplasm of *C*. *reinhardtii* after exposure to AgNPs via the EDX spectra (Fig. 6C). Although the EDX spectral signal clearly showed Ag in the cytoplasm of *C*. *reinhardtii* after exposure to AgNPs, it is uncertain if the source of Ag was from the uptake of AgNPs or from Ag ions released from the AgNPs. Additionally, EDX images showed that Ag and S nearly overlapped one another in the cytoplasm of *C*. *reinhardtii* after exposure to AgNPs (Fig. 6B). In agreement with our findings, Wang *et al*.^51^ verified the co-existence of Ag with S in the cytoplasm of *C*. *reinhardtii* after exposure to pvp-AgNPs (7–17 nm) using high-resolution secondary ion mass spectrometry. These authors reported that Ag in the cytoplasm was attributed to Ag ions released from AgNPs, based on the presence of AgNP sulfidation products (e.g., silver sulfide and silver thiolate) using selected area electron diffraction and X-ray absorption spectroscopy. In addition, Leclerc *et al*.^52^ reported the presence of Ag in the cytoplasm of *C*. *reinhardtii* after exposure to polyacrylate-coated AgNPs (2–11 nm), based on a reduction or the precipitation of Ag ions. In the present study, based on the co-existence of Ag with S in the cytoplasm (as confirmed by EDX imaging), it is possible that Ag ions were released from the AgNPs. However, more research on the source of Ag in algal cells is needed, as the exact source of Ag observed in the cytoplasm of *C*. *reinhardtii* in the present study was not confirmed.

### Effects of AgNP-soils on soil algal membrane permeability, esterase activity, and oxidative stress

**Figure 7:**
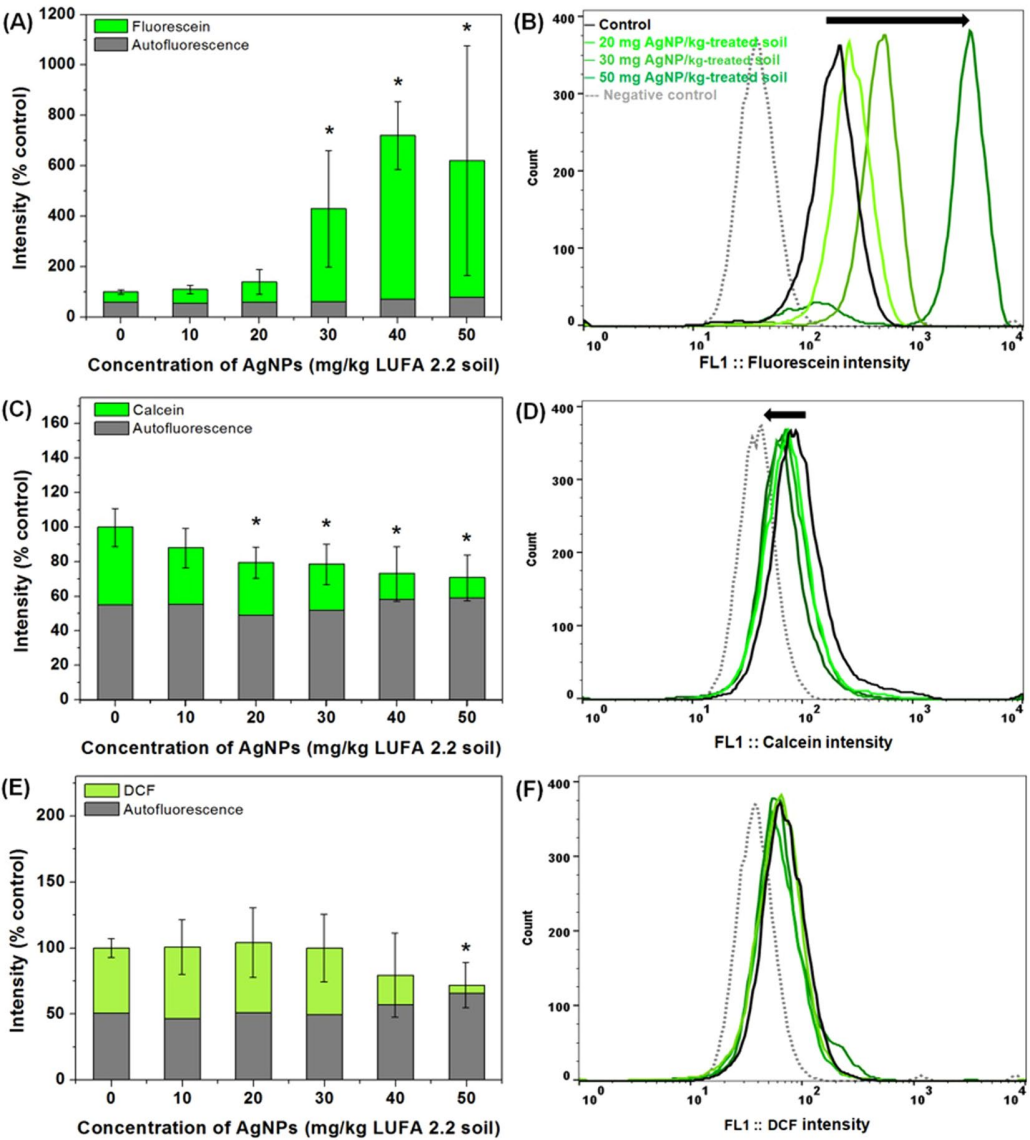
Cell membrane permeability (**A** and **B**), esterase activity (**C** and **D**), and oxidative stress (**E** and **F**) of *Chlamydomonas reinhardtii* after exposure to silver nanoparticles (AgNPs) for six days. Bars represent the standard deviation of the mean (n = 6–12). Asterisks (*) indicate results that are significantly different from those of the control (*p* < 0.05).

The effects of AgNP-treated soils on membrane permeability, esterase activity, and oxidative stress in *C*. *reinhardtii* after FDA, calcein-AM, and DCFH-DA staining can be seen in Fig. 7. Although FDA is first hydrolyzed to fluorescein by esterase and then retained as green fluorescent fluorescein, cell permeability can be enhanced when the integrity of cell membranes have been affected by chemicals^31^. In the present study, we observed that fluorescein intensity (indicating cell membrane permeability) increased with increasing concentrations of AgNPs (Fig. 7A,B). Cell membrane permeability was 4.3, 7.2, and 6.21-fold higher than that of control cells in 30, 40, and 50 mg AgNP/kg-treated soil, respectively. Similarly, the penetration of AgNPs into the cytoplasm and disturbances in cellular functions have been reported to occur after an increase in the permeability of bacterial cell membranes^53,54^. Calcein-AM is hydrolyzed and converted to green fluorescent calcein by esterase, and therefore calcein intensity is indicative of cellular esterase activity or cell viability^28,32^. As shown in Fig. 7C, esterase activity (calcein intensity) was significantly inhibited at ≥ 20 mg AgNP/kg-treated soil (*p* < 0.05) when *C*. *reinhardtii* was exposed to AgNPs for six days. Compared to controls, esterase activity was 88%, 80%, 79%, 73%, and 71% at exposure concentrations of 20–50 mg AgNP/kg-treated soil. It is generally accepted that DCFH-DA is oxidized and converted to dichlorofluorescin (green fluorescence), and is therefore used as an indicator of oxidative stress^33^. In addition, it has been previously reported that AgNPs caused oxidative stress in two algae (*Chlorella vulgaris* and *Dunaliella tertiolecta*) in AgNP-treated liquid media^55^, in the earthworm *Eisenia fetida* in AgNP-treated soil^56^, and in the nematode *Caenorhabditis elegans* in AgNP-treated liquid media^57^. In contrast, a decrease in the intensity of DCF was observed in response to 40 and 50 mg AgNP/kg-treated soil in the present study (Fig. 7E), which could be related to the observed increase in the mucilaginous sheath in response to increasing concentrations of AgNPs. Interestingly, the differences between green autofluorescence intensity and calcein and dichlorofluorescin were only 12–15% and 6–22%, respectively, in 40 and 50 mg AgNP/kg-treated soils. The representative histograms from flow cytometry exhibit similar intensities between calcein-AM stained and unstained autofluorescence (Fig. S5A), and between DCFH-DA stained and unstained autofluorescence (Fig. S5B). These results suggest that *C*. *reinhardtii* exposed to 40 and 50 mg AgNP/kg-treated soils were not fully stained by calcein-AM or DCFH-DA, likely owing to the formation of mucilaginous sheaths, as evidenced by increased FSC signals (Figs 3, 4D and F). Therefore, we could not completely confirm significant esterase activity and oxidative stress in 40 and 50 mg AgNP/kg-treated soils as a result of the inconsistent penetration of dyes into the mucilaginous sheath, and thus inconsistent staining with calcein-AM or DCFH-DA after the formation of mucilaginous sheaths induced by AgNPs.

### Effects of bulk Ag or Ag ions on algal biomass, cell size, cell granularity, and membrane permeability

Biomass, cell size, cell granularity, and membrane permeability of *C*. *reinhardtii* in response to bulk Ag and Ag ions are shown in Figures S6 and S7, respectively. We were not able to analyze data from flow cytometry experiments on 40 and 50 mg Ag ion/kg-treated soil as a result of low cell density. *Chlamydomonas reinhardtii* exhibited no significant response of cell granularity to Ag bulk- or ion-treated soils (Figs S6B and S7B), as was observed when exposed to AgNP-treated soils. *Chlamydomonas reinhardtii* showed no significant changes in biomass in response to Ag bulk-treated soils (Fig. S6A), unlike the significant changes in biomass observed in response to AgNPs-treated soils. However, *C*. *reinhardtii* did exhibit a significant increase in cell size (Fig. S6B) and membrane permeability (Fig. S6C) in Ag bulk-treated soils, as observed in response to AgNPs-treated soils. In response to Ag ion-treated soils, *C*. *reinhardtii* exhibited a decrease in both biomass (Fig. S7A) and cell size (Fig. S7B), and an increase in membrane permeability (Fig. S7C), as observed in response to AgNPs-treated soils, with the exception of the decrease of cell size. The response of *C*. *reinhardtii* to Ag bulk-treated soils may be related to the adsorption of Ag bulk onto *C*. *reinhardtii* in Ag bulk-treated soils, or an increase in the quantity of vegetative cells (as evidenced by the general increase in biomass) in Ag bulk-treated soils, and a decrease in the quantity of vegetative cells in Ag ion-treated soils. Although we observed a mucilaginous sheath in *C*. *reinhardtii* adsorbed to soil after exposure to Ag bulk and Ag ions for 6 d, we did not observe a mucilaginous sheath in *C*. *reinhardtii* suspended in the 50 mg Ag bulk/kg-treated soil extracts and 30 mg Ag ion/kg-treated soil extracts (Fig. S8). This phenomenon may be related to defense a mechanism of *C*. *reinhardtii* adsorbed to soil owing to desiccation, and not the toxicity of Ag bulk or Ag ions. With these results, we concluded that Ag ions were the most toxic of all Ag materials tested, followed by AgNPs, and then Ag bulk for *C*. *reinhardtii* in soil. As observed with *C*. *reinhardtii* in the present study, the inhibition of growth was observed in the freshwater alga *Pseudokirchneriella subcapitata*, the marine alga *Phaeodactylum tricornutum*, and the water flea *Ceriodaphnia dubia*. In addition, changes in the levels of reactive oxygen species, catalase activity, DNA damage, acid phosphatase activity, multixenobiotic resistance transport activity, and Na-K-ATPase activity in the mussel *Mytilus galloprovincialis* were induced by exposure to Ag materials in the following order: Ag ions > PVP-coated AgNPs > micro-sized Ag particles^58,59^. They reported the mechanism of toxicity to be the relative surface area and Ag ions dissolved from the Ag nano. In this study, there was no significant dissolution of Ag ions from the AgNP-treated soil and mucilaginous sheath of *C*. *reinhardtii* suspended in the 50 mg Ag bulk/kg-treated and 30 mg Ag ion/kg-treated soil extracts. Sensitivity to the toxicity of Ag ions was higher, while the sensitivity to the toxicity of Ag bulk was lower than sensitivity to AgNPs. Therefore, we concluded that the toxicity of *C*. *reinhardtii* in soil treated with AgNPs depends solely on particle (nano) size, and not to Ag in general.

## Conclusions

In conclusion, there are several measurable effects of AgNPs on the soil alga *C*. *reinhardtii* in soil media: (i) the production of mucilaginous sheath, (ii) the inhibition of biomass, (iii) the partial inhibition of photosynthetic activity, (iv) an increase in cell size, (v) an increase in membrane permeability, and (vi) the presence of Ag inside algal cells. As the production and consumption of AgNPs continue to rise, it is important to elucidate the potential effects of the continued release of AgNPs into the environment. Thus, the present study demonstrates a novel and effective method for evaluating the toxicity of nanomaterials to soil algae.

## Electronic supplementary material

Supplementary Information
