# Supplementary material for: Quantification of silver nanoparticle toxicity to algae in soil via photosynthetic and flow-cytometric analyses
Source: Sci Rep. 2018 Jan 10;8:292. doi: 10.1038/s41598-017-18680-5 (PMC5762909; doi:10.1038/s41598-017-18680-5)

Supporting Information

Quantification of silver nanoparticle toxicity to algae in soil via photosynthetic and flow-cytometric analyses

Sun-Hwa Nam, Jin Il Kwak, and Youn-Joo An*

Department of Environmental Health Science, Konkuk University, 120 Neungdong-ro, Gwangjin-gu, Seoul 05029, Korea

*Corresponding author
Tel: +82 2 2049 6090
Fax: +82 2 2201 6295
E-mail: anyjoo@konkuk.ac.kr

Table S1. Physicochemical properties of test soil

Physicochemical properties		LUFA 2.2 soil
Texture		Loamy sand
pH		5.6
WHC (mL/g)		0.54
Available phosphate (mg/kg)		24
Total nitrogen (mg/kg)		375
Organic matter (%)		3.39
Exchangeable cations (Cmol$^+$ /kg)	Ca	9.43
	K	0.18
	Mg	0.4
Total metals (mg/kg)	As	11.24
	Cd	Not detected
	Cu	2.41
	Ni	2.61
	Pb	19.17
	Zn	16.36

Fig. S1. Dot plots of collected supernatant used to distinguish pure *Chlamydomonas reinhardtii* population and unspecific debris. (A) Un-inoculated sample and (B) *C. reinhardtii*-inoculated sample. The gates are defined as the region of pure *C. reinhardtii* population used for statistical analysis. The outer gates are defined as the region of unspecific debris.

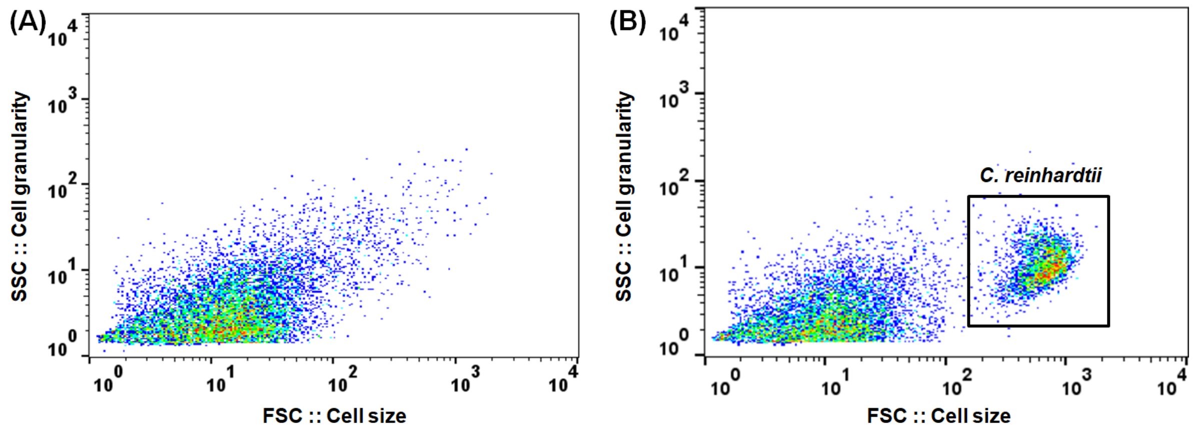

Fig. S2. Field emission transmission electronic micrograph (FE-TEM) of silver nanoparticles (AgNPs).

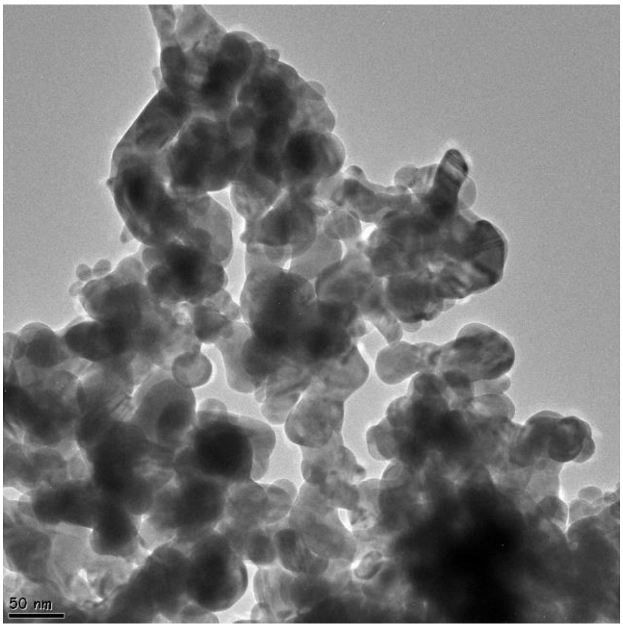

Fig. S3. High-resolution scanning electron microscopy (HR-SEM) images of silver nanoparticles (AgNPs) in the tested soil at 50 mg AgNPs/kg (dry weight). Adsorption of AgNPs on the soil surface was evaluated by energy-dispersive X-ray spectroscopy detector (EDX). (A–D) show HR-SEM images and (E-H) show EDX spectra of an electron dense spot. The red circles indicate the presence of AgNPs on the soil surface, whereas blue circles indicate absence of AgNPs in the normal soil.

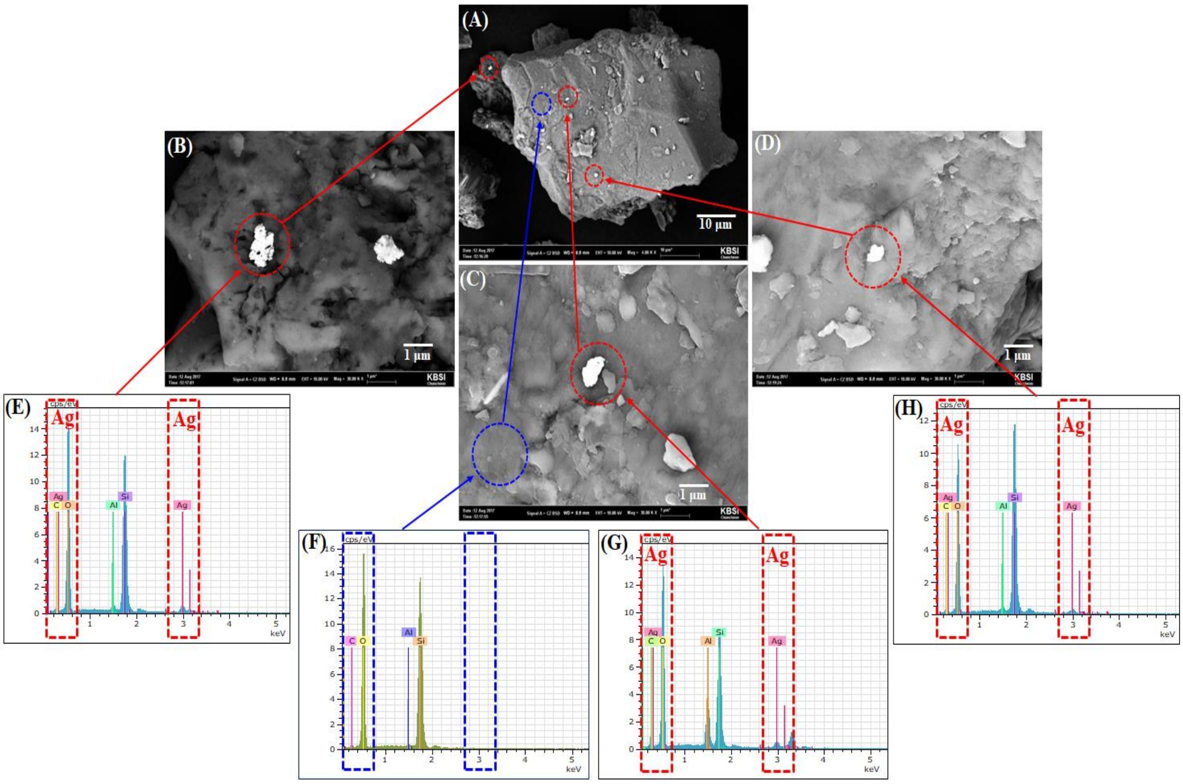

Fig. S4. Field emission transmission electron micrographs (FE-TEM) and energy-dispersive X-ray spectroscopy detector (EDX) images of *Chlamydomonas reinhardtii* in untreated soil. (A) FE-TEM (B) EDX images show the elemental distribution of Ag, S, C, N, and O in the red dotted square outlining the expanded FE-TEM micrograph from panel A. (C) EDX spectrum shows the elemental analysis (C, N, and O) in blue circles above the yellow peaks. Yellow dotted rectangle indicates the absence of Ag in *C. reinhardtii*.

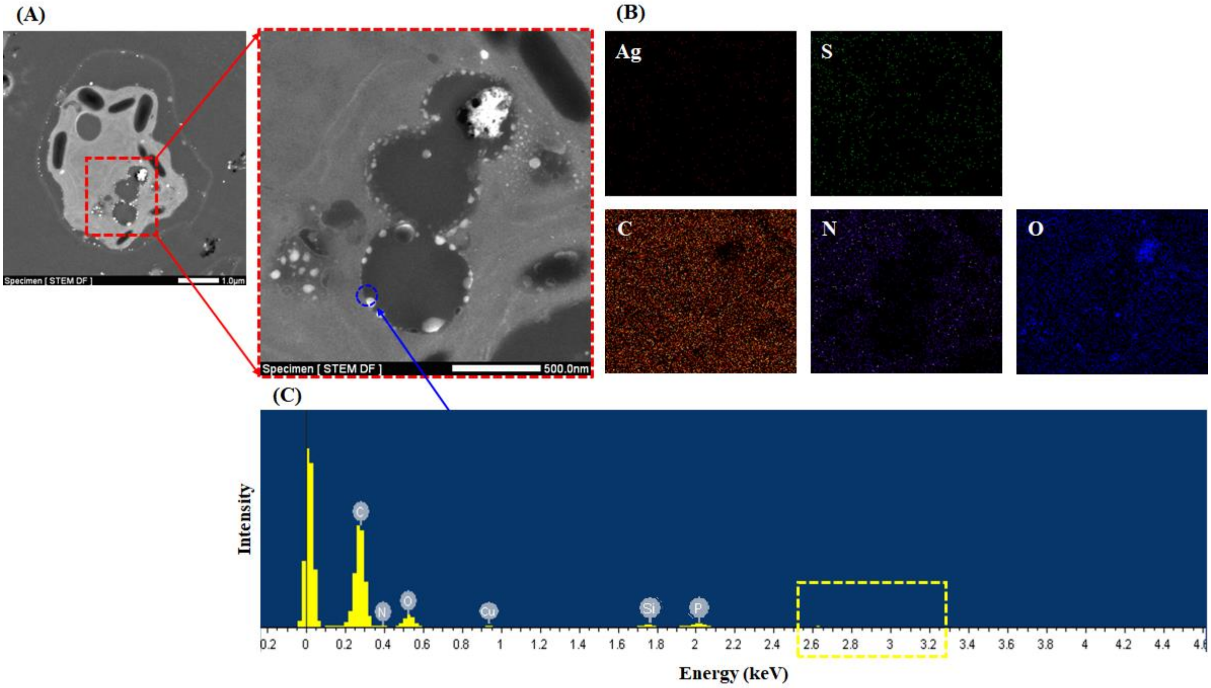

Fig. S5. Representative flow cytometric histogram for (A) Calcein-AM staining and unstaining (esterase activity), and (B) DCFH-DA staining and unstaining (oxidative stress) of *Chlamydomonas reinhardtii* after exposure to silver nanoparticles (AgNPs) for six days at maximum concentration. Dashed grey lines indicate unstained algae in maximum exposure groups (50 mg/kg dry soil), and dark green lines indicate algae stained with (A) calcein-AM or (B) DCFH-DA in maximum exposure groups (50 mg/kg dry soil). At the maximum exposure concentration, stained or unstained histograms of algae mostly overlapped compared to lower exposure concentration (described in Fig. 7). These results indicated that algae could not be stained with calcein-AM and DCFH-DA after 6d-AgNP exposure at maximum concentration (50 mg/kg dry soil).

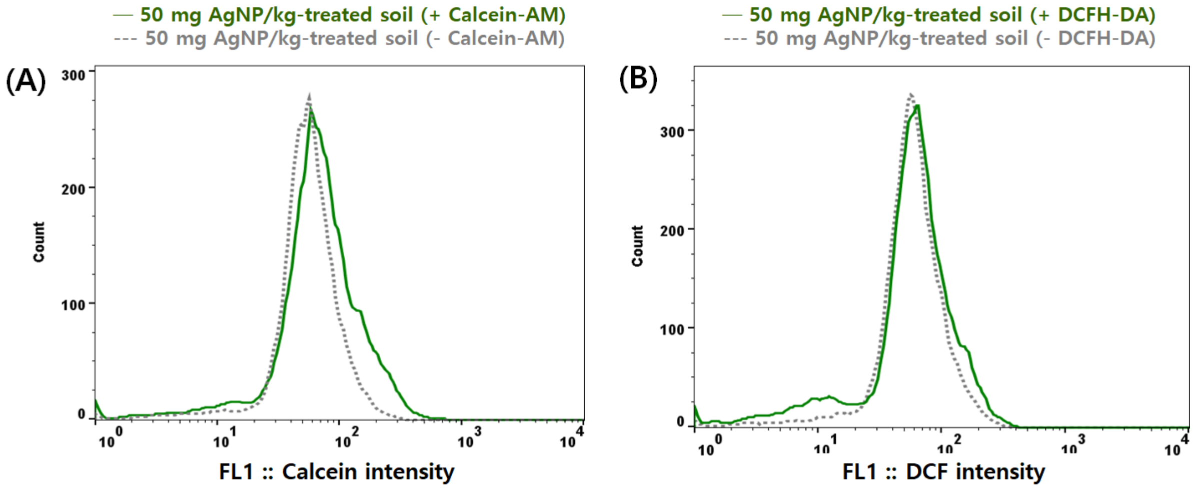

Fig. S6. Effects of *Chlamydomonas reinhardtii* after exposure to Ag bulk for six days. Bars represent the standard deviation of the mean of three replicates. Asterisks (*) indicate significantly different values in relation to those obtained in the control (p <0.05).

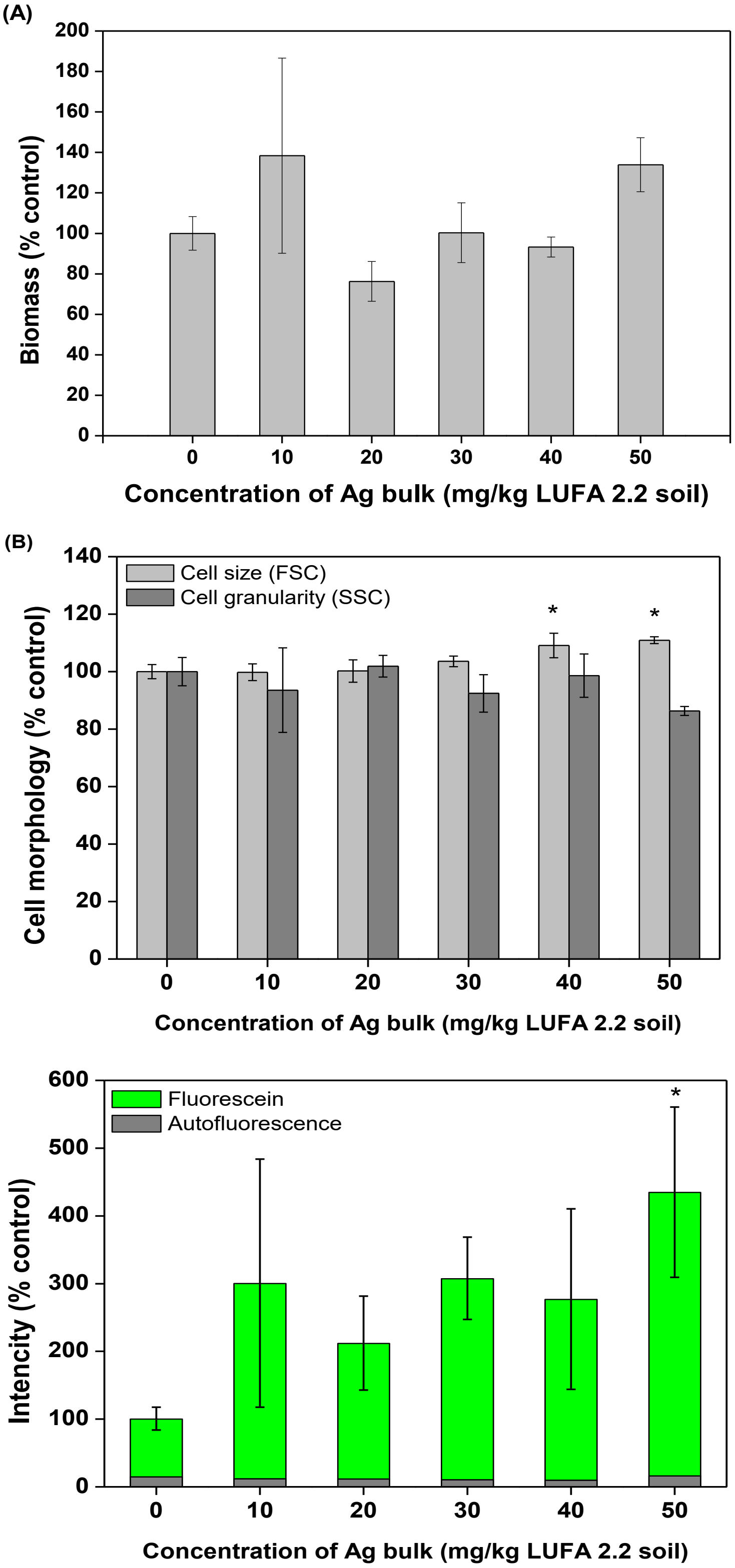

Fig. S7. Effects of *Chlamydomonas reinhardtii* after exposure to Ag ions for six days. Bars represent the standard deviation of the mean of three replicates. Asterisks (*) indicate significantly different values in relation to those obtained in the control ($p < 0.05$).

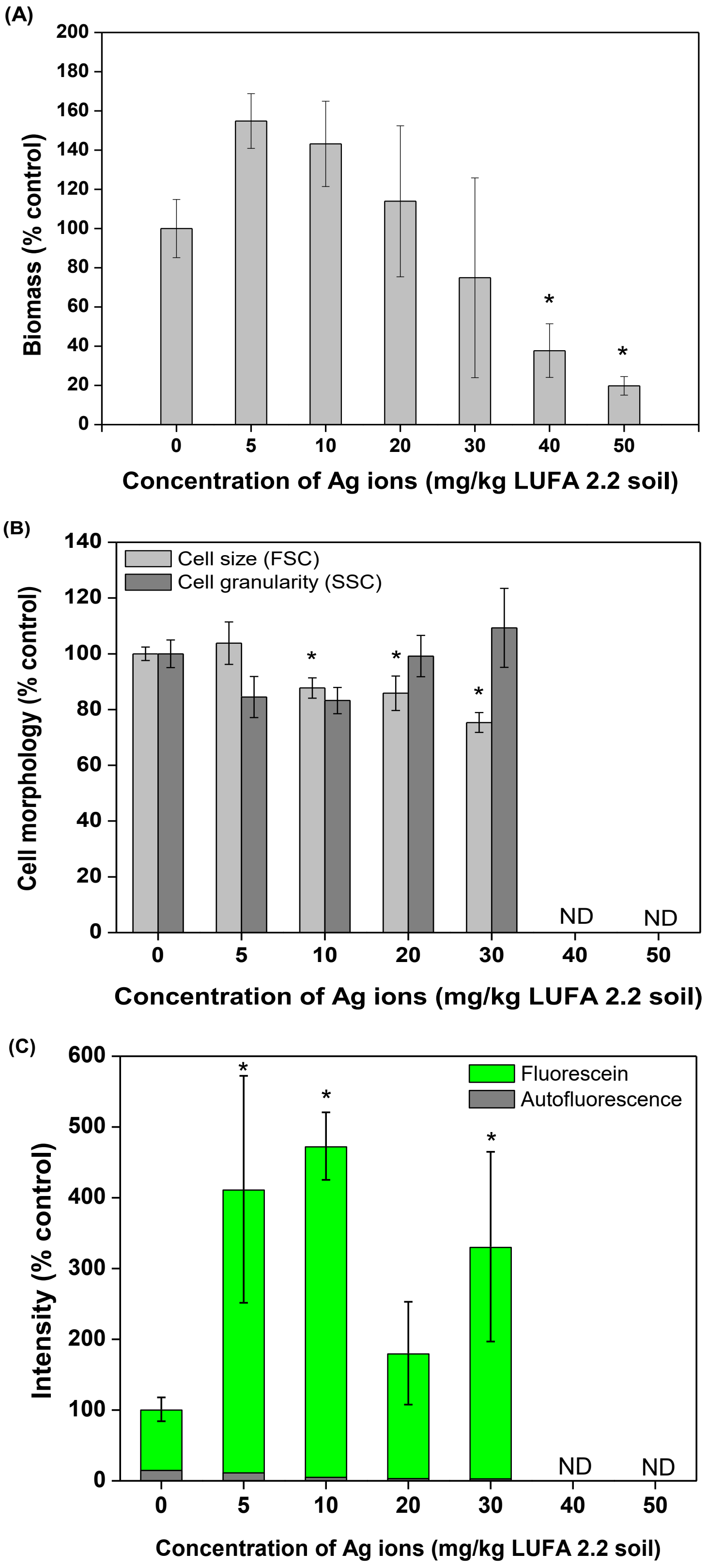

Fig. S8. Light microscopy images of *Chlamydomonas reinhardtii* after exposure to Ag bulk and ions for six days. (A) *C. reinhardtii* adsorbed to the untreated soil, (B) *C. reinhardtii* suspended in the untreated soil extracts, (C) *C. reinhardtii* adsorbed to the 50 mg Ag bulk/kg-treated soil, (D) *C. reinhardtii* suspended in the 50 mg bulk/kg-treated soil extracts, (E) *C. reinhardtii* adsorbed to the 30 mg Ag ions/kg-treated soil, and (F) *C. reinhardtii* suspended in the 30 mg Ag ions/kg-treated soil extracts. Black arrow means soil particles. Blue arrows mean mucilaginous sheath.

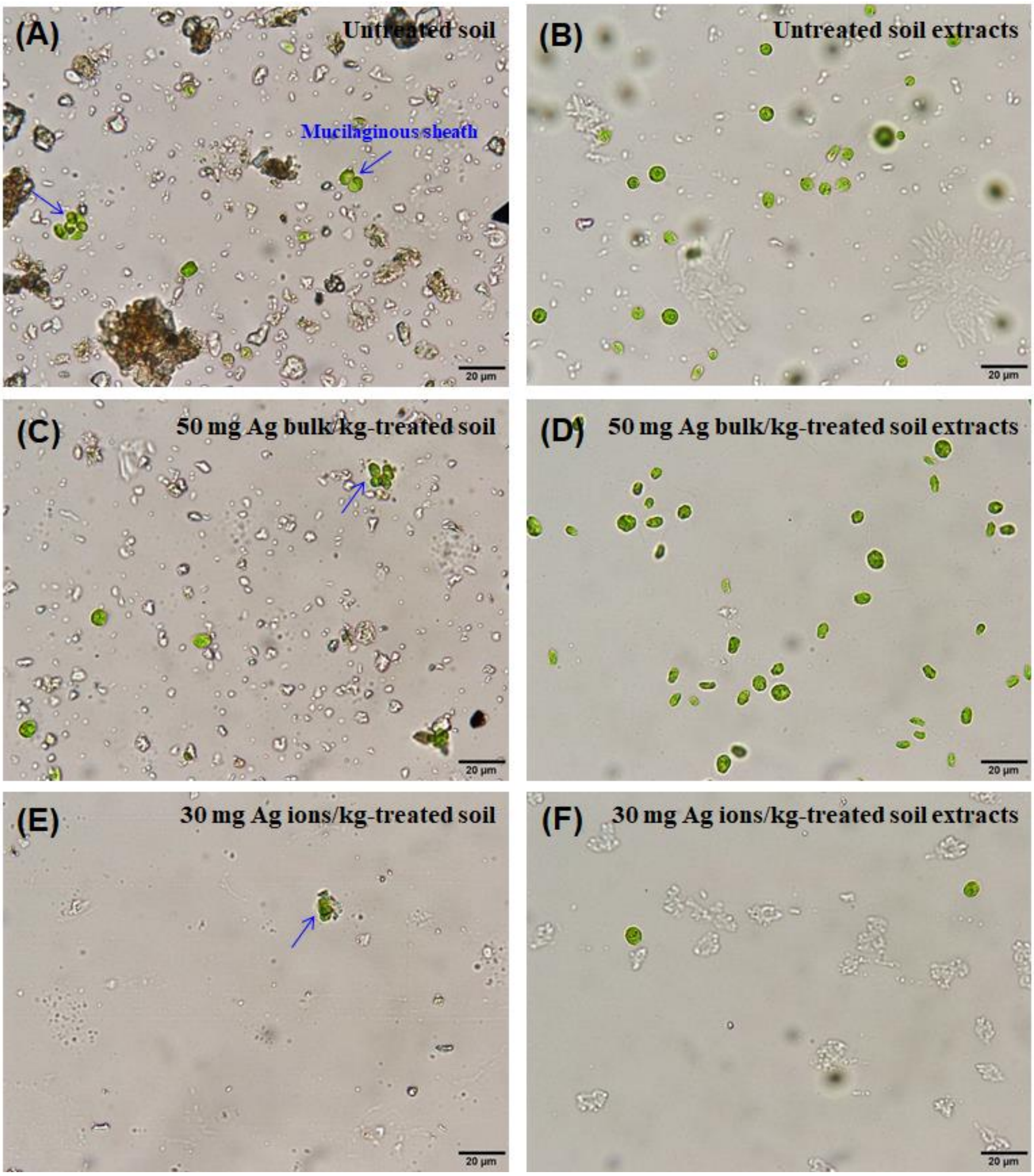

Supplement: Supplementary file 1 — Supplementary Information [file 41598_2017_18680_MOESM1_ESM.pdf]
